# Structural dynamics of GluK2 kainate receptors in apo and partial agonist bound states

**DOI:** 10.21203/rs.3.rs-3592604/v1

**Published:** 2023-12-02

**Authors:** Nebojša Bogdanović, Guadalupe Segura-Covarrubias, Lisa Zhang, Nami Tajima

**Affiliations:** 1 Department of Physiology and Biophysics, Case Western Reserve University School of Medicine, Ohio, 44106, USA.; 2 Equal contribution

**Keywords:** Kainate-type ionotropic glutamate receptsors, structure, gating mechanism, partial agonist, cryo-electron microscopy, patch-clamp electrophysiology, *N*-glycans

## Abstract

Kainate receptors (KARs) belong to the family of ionotropic glutamate receptors (iGluRs) and are tetrameric ligand-gated ion channels that regulate neurotransmitter release and excitatory synaptic transmission in the central nervous system. While KARs share overall architectures with other iGluR subfamilies, their dynamics are significantly different from those of other iGluRs. KARs are activated by both full and partial agonists. While there is less efficacy with partial agonists than with full agonists, the detailed mechanism has remained elusive. Here, we used cryo-electron microscopy to determine the structures of homomeric rat GluK2 KARs in the absence of ligands (apo) and in complex with a partial agonist. Intriguingly, the apo state KARs were captured in desensitized conformation. This structure confirms the KAR desensitization prior to activation. Structures of KARs complexed to the partial agonist domoate populate in domoate bound desensitized and non-active/non-desensitized states. These previously unseen intermediate structures highlight the molecular mechanism of partial agonism in KARs. Additionally, we show how *N*-glycans stabilized the ligand-binding domain dimer via cation/anion binding and modulated receptor gating properties using electrophysiology. Our findings provide vital structural and functional insights into the unique KAR gating mechanisms.

## Introduction

Kainate receptors (KARs), together with α-amino-3-hydroxy-5-methylisoxazole-4-propionate receptors (AMPARs), N-methyl-D-aspartate receptors (NMDARs), and delta receptors constitute the ionotropic glutamate receptor (iGluR) superfamily. They are ligand-gated ion channels ubiquitously expressed in the central nervous system and mediate excitatory synaptic transmission and plasticity^1,2^. KARs are involved in high-level cognitive functions, such as learning and memory, and are also associated with various brain diseases^3^, including epilepsy^4,5^ and schizophrenia^6–9^. They are expressed both pre- and postsynaptically^10^. Postsynaptic KARs mediate synaptic transmission with other glutamate receptors^11^, while presynaptic KARs regulate neurotransmitter release and modulate network excitability^10^. KARs are cation-permeable receptors composed of five subunits: GluK1 to GluK5. GluK1–GluK3 can form homotetramers, while GluK4–GluK5 only form functional heterotetramers in combination with GluK1–GluK3^10^. KAR subunits comprise four domains: amino-terminal domain (ATD), ligand binding domain (LBD), transmembrane domain (TMD), and intracellular C-terminal domain (CTD). The function of KARs is controlled by various ligands, including agonists, competitive antagonists, allosteric modulators, and channel blockers^12–14^. Recent cryo-electron microscopy (cryo-EM) studies have revealed the overall architectures of the KAR subfamily^15–21^, mainly in agonist-, antagonist- and allosteric modulator-bound desensitized, inhibited, or non-active states. Together with crystal structures of isolated ATD or LBD of KARs in complex with various ligands provided important structural information, including the ligand binding modes and the conformational alternation upon ligand binding on the receptors^22^. In particular, a key feature of KARs demonstrated earlier is that KARs undergo large structural changes with the LBD dimer assembly dissociation upon desensitization, which is not observed in non-KAR iGluRs^1,21,23–26^. The LBD dimer dissociation with the large in-plane rotation (~110°) of the subunit BD results in LBDs forming nearly four-fold symmetric conformation viewing from the top while other iGluR subfamilies in desensitized states maintain their two-fold symmetric arrangement. These unusual dynamics implicate the unique gating mechanism of KARs. However, the function-dependent conformational transitions and gating mechanism of KARs remain unclear, largely due to the lack of structural information on active-state KARs, as compared to the wealth of information available on AMPARs and NMDARs.

While various agonists can activate KARs, not all elicit maximal responses. Compared to full agonists, partial agonists yield submaximal responses due to their lower intrinsic activity at KARs. Despite displaying high affinity, partial agonists tend to exhibit less efficiency and slower channel opening. Previous electrophysiological studies have shown the efficacy spectrum of partial agonists, which indicates that their receptors have multiple inactive (agonist-bound, channel-closed) intermediate states toward channel opening, presumably different from the transition pathway of receptors elicited by full agonists^27^. Additionally, other studies have suggested that partial agonists generate different conformational changes and undergo distinct transitions during desensitization^28^. Specifically, a recent study showed the high-potency KAR partial agonist, (2S,3S,4S)-3-carboxymethyl-4-[(1Z,3E,5R)-5-carboxy-1-methyl-hexa-1,3-dienyl]-pyrrolidine-2-carboxylicb acid (domoate), suppresses hyperexcitation by selectively activating KARs expressed in GABAergic neurons, indicating a potential approach to treating brain diseases using domoate, including epilepsy^29^.

In this study, we first demonstrate the cryo-EM structures of full-length GluK2 KARs in apo and in complex with domoate that address the mechanistic understanding of KARs controlled by domoate. Furthermore, we show how the *N*-glycans stabilize the LBD dimer and modulates KAR gating.

## Results

### Functional analysis and purification

To understand the gating mechanism of KARs, we investigated the structure of homotetrameric GluK2 KARs. To obtain high-quality stable tetrameric receptors, we used the previously described rat GluK2 mutant construct^16,19^. The GluK2 gene was RNA-edited at residue position 567 (I to V), and the construct contained two mutations, C576V and C595S, and a C-terminal twin strep tag. We first analyzed the functional properties of the wild-type GluK2 (GluK2 WT) and cryo-EM (GluK2 EM) constructs using whole-cell patch clamp electrophysiology. The recombinant rat GluK2 WT and EM constructs were expressed in HEK293 cells and activated by 1 s or 3 ms application of saturated (10 mM) glutamate to analyze the desensitization and deactivation time constants (τ_desen_ and τ_deact_, respectively). As shown in Fig. S1A-D and Table S1, the GluK2 WT construct show rapid desensitization and deactivation in response to a prolonged application of glutamate as well as high EC_50_. The GluK2 EM construct displayed similar properties as the GluK2 WT, therefore, we concluded that the cryo-EM construct retains the basic functional properties similar to the GluK2 WT construct. We next expressed GluK2 EM in *Spodoptera frugiperda* (Sf9) insect cells using the EarlyBac method^30^. We successfully purified the tetrameric receptors using Strep-Tactin Sepharose in a detergent, n-dodecyl-β-D-maltoside (DDM) (see the Method section). The purified sample showed a single symmetrical peak in Superose-6 size exclusion chromatography, and the peak fraction was subjected to single-particle cryo-EM (Fig. S1E–G).

### Cryo-EM structure of intact rat GluK2 in resting state

To identify the overall conformational changes upon agonist binding, we first obtained the apo state structure of GluK2 KAR by removing residual glutamate from the insect cells through extensive washing during the Strep-tactin Sepharose purification step. We first refined the structure in C1 symmetry and analyzed the overall conformation of each chain (Fig. S2). The 3D classification resulted in three classes, with the major class appearing as two-fold symmetrical. Thus, C2 symmetry was imposed during the 3D refinement step, which resulted in a structure with an overall resolution of 3.0 Å, as estimated by the 0.143 FSC gold standard (Fig. S3, Table S1). While we did not pursue further, two other classes showed a similar overall structure as the high-resolution apo class we used. Currently, there is no structure of isolated GluK2 LBD in the apo state, likely due to its protein conformational flexibility^22^. Additionally, the previously determined cryo-EM structures of the intact homo- and heterotetrameric KARs in apo states ^18,19^ have limited resolutions. Consequently, it is challenging to conduct detailed analyses of the conformation of each domain and evaluate the presence or absence of ligands in the binding pocket^18,19^. This 3.0 Å structure in the apo state overcomes the abovementioned limitation.

**Figure 1.**
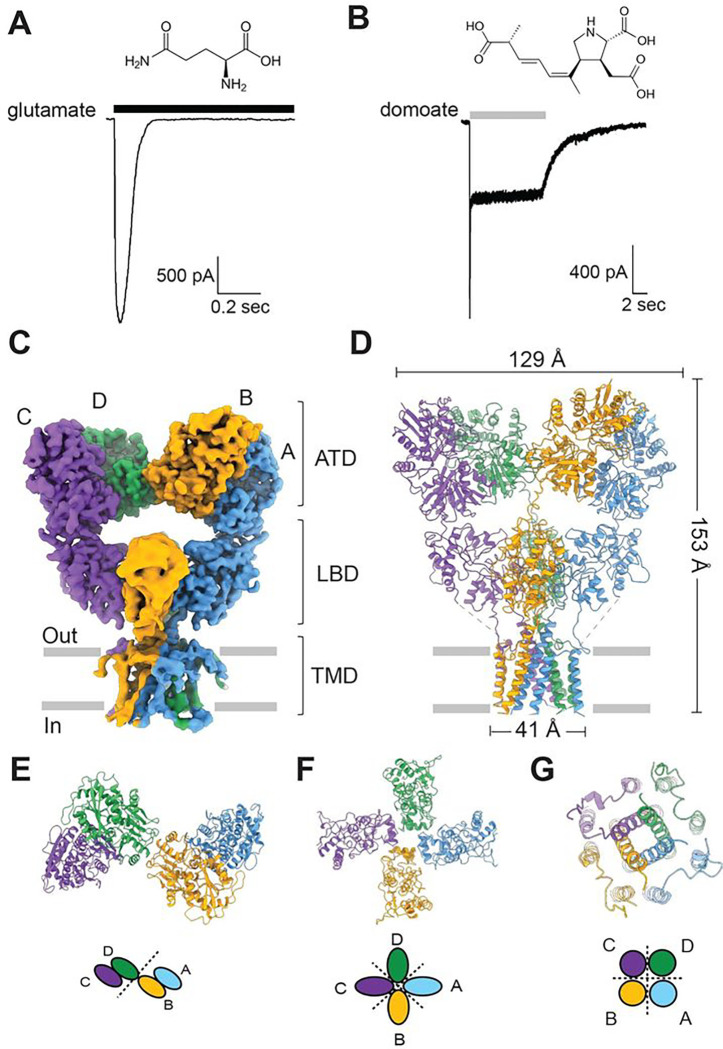
GluK2 KAR structure and function. **(A, B**) Top: The chemical structures of L-glutamate and domoate. Bottom: Representative whole-cell currents recorded at −70 mV holding voltage from HEK 293 cells expressing rat GluK2 WT in response to 1 s application of L-glutamate or 10 s application of domoate. **(C)** Cryo-EM map of the tetrameric GluK2 KAR in an apo state viewed perpendicular to the membrane. The A–D chains are colored in cyan, yellow, green, and purple, respectively. This panel shows the EM map for ATD, LBD, and TMD layers. **(D)** Graphic representation of the GluK2 model in the apo state colored as in (C). **(E–G)** Three domains viewed from the extracellular space, showing approximately two-fold symmetry of ATD and four-fold local symmetries of the LBD and TMD layers of the ligand-free apo state of GluK2 KAR.

**Figure 2.**
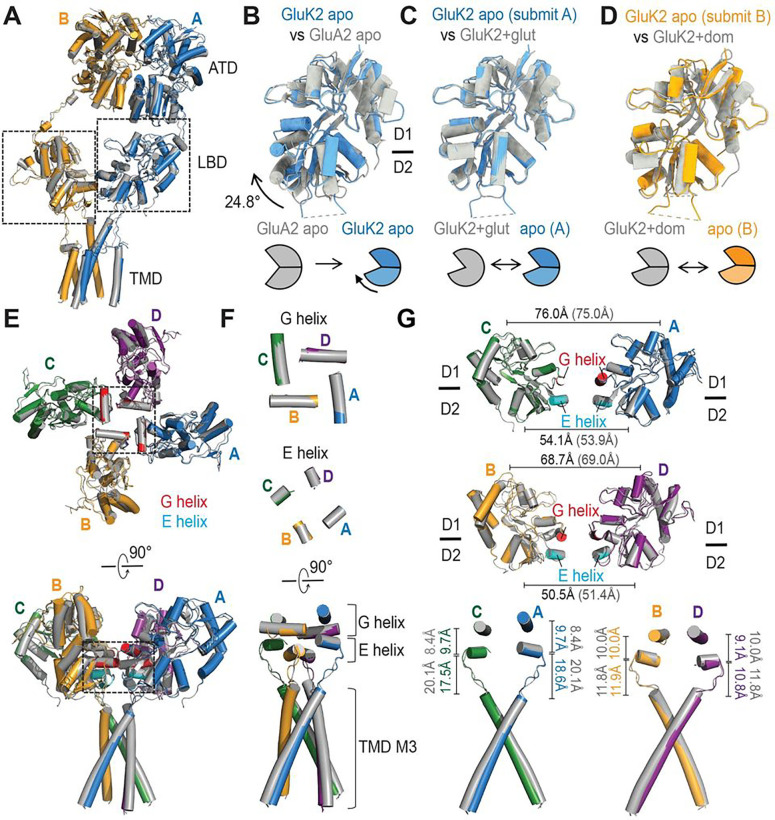
Structural analysis of ligand-free apo-state GluK2. **(A)** Subunits A and B of the apo state GluK2 structure with the A and B chains colored in cyan and yellow respectively, aligned to the glutamate bound GluK2 structure (PDB ID: 5KUF) in gray. LBDs are highlighted. **(B–D**) The GluK2 LBD structures of subunits A and B in the ligand-free apo state in cyan and yellow compared to the crystal structure of isolated LBD of GluA2 in the apo state (PDB ID: 1FTO), glutamate bound GluK2 LBD (PDB ID: 3GTF), and domoate bound GluK2 LBD (PDB ID: 1YAE) in gray. The degree of LBD bi-lobe closure is measured and indicated. Depicted is the LBD bi-lobe closing in subunit A of apo GluK2 KAR compared to the apo GluA2 AMPAR (left), subunit A and glutamate bound GluK2 KAR (middle), and subunit B and domoate bound GluK2. The GluK2 LBDs of subunits A and B in the absence of ligands are stabilized in bi-lobe closed conformations similar to the glutamate and domoate bound crystal structures of isolated GluK2 LBD, respectively. **(E**) The top view of the LBD layer, and the side view of LBD-TMD layers for GluK2 apo state conformation aligned to the glutamate bound desensitized GluK2 (PDB ID: 5KUF) structure in gray. G and E helices are colored in red and cyan, respectively. The desensitization rings are highlighted. **(F**) Top view of the G and E helices, and the side view of the desensitization ring consisting of the G and E helices with the TMD M3 helices. **(G**) Side view of the LBD–TMD layers. Top and middle: subunit AC and BD LBD pairs shown as viewed from the side with the distances between the center of masses (COMs) of D1 or D2 lobes of the subunit AC pair and the subunit BD pair for the apo and glutamate bound GluK2. Bottom: Shown are the distances between the COMs of the E helices and the top of the TMD M3 helices for the apo and glutamate bound GluK2 viewed from the side.

The apo state structure shows three layers—ATD, LBD, and TMD—consistent with the previously determined structures (Fig. 1C, D). Each ATD exhibits a classical bi-lobe architecture comprising upper and lower lobes (R1 and R2, respectively), superposable over the crystal structure of isolated GluK2 ATD (PDB ID: 3H6G) with an Root Mean Square Deviation (RMSD) of 0.9–1.1 Å. As previously shown, the subunit AB and CD pairs are arranged as dimers (Fig. 1D, E). The ATD layer containing two dimers showed only a minor conformational difference compared to the glutamate bound GluK2 KAR structure (PDB ID: 5KUF), with minimal contact between ATD and LBD (Fig. 2A). Furthermore, each LBD forms a typical clamshell-like conformation with the upper lobe (D1) and lower lobe (D2) (Fig. 2B–D). To confirm that this is an apo state structure with no ligand contamination, we first analyzed the agonist occupancy in the LBD bi-lobes. This apo GluK2 structure solved at 3.0 Å with no obvious agonist density within the agonist binding pockets of all four subunits indicates that most of the receptors in this state do not contain agonists (Fig. S4 a–d). Interestingly, all four LBDs in this structure, in the absence of ligands, were stabilized in closed-cleft conformations, in contrast to the apo state structures of AMPAR and NMDAR LBDs stabilized in open-cleft conformations (Fig. 2B). The LBD bi-lobes in the subunit AC pair are tightly closed, similar to the glutamate bound GluK2 LBD structure (PDB ID: 3G3F) (Fig. 2C). Although the LBD bi-lobes in the subunit BD pair are also closed, they are stabilized rather similarly to the domoate bound GluK2 LBD conformation (PDB ID: 1YAE) with the clamshell 11.2–11.7° more open than the subunit AC pair ^31,32^ (Fig. 2D).

**Figure 3.**
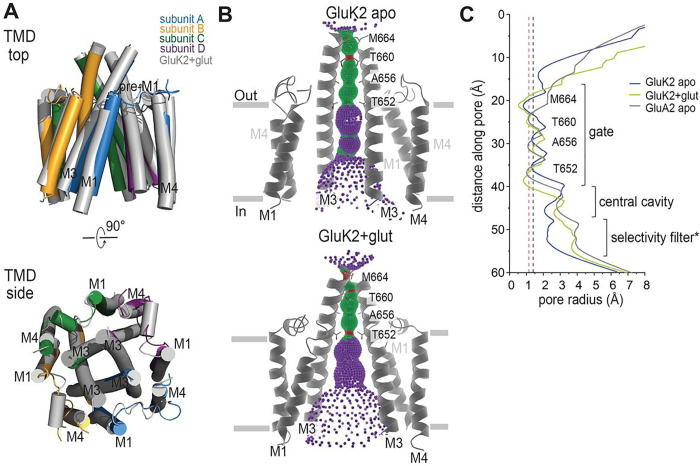
Ion channel structure of apo state GluK2 KARs. **(A)** The TMD layer of apo GluK2 aligned to the glutamate bound GluK2 (PDB ID: 5KUF), as seen parallel to the membrane (top) and perpendicular to the membrane from the extracellular side. **(B)** The pore profile of the apo GluK2 KAR and the glutamate bound GluK2, showing M3 helices and their pore-lining residues for the two subunits. The pore radius was calculated using HOLE. Regions with a pore radius less than 1.15 Å are colored red, water-accessible sections with a radius larger than 1.15 Å and less than 2.5 Å are colored green, and sections with a radius larger than 2.5 Å are colored purple. **(C)** Pore diameter comparison between the unliganded apo GluK2 KAR, glutamate bound desensitized state of GluK2, and the unliganded apo GluA2 AMPAR (PDB ID: 4U2P).

In addition to the LBD clamshell opening degree, the LBD arrangement is notably distinct from the previously predicted conformation^33^. The overall architecture of our apo state structure is similar to that of the glutamate bound desensitized GluK2 structure (PDB ID: 5KUF), with an RMSD of 1.53 Å, except for some minor local conformational differences mainly in the D1 domains of LBD in the B–D subunit (Fig. 2A). The LBDs in the ligand-free apo GluK2 KAR structure are arranged in approximately four-fold symmetry viewing from the top like the glutamate bound structure (Fig. 1E–G), in contrast to the expected two-fold symmetric arrangement (four LBDs forming dimer-of-dimers). As expected from the extracellular domain organization, the TMD of our apo state structure also shows a similar geometry to the glutamate bound GluK2 KARs (Fig. 3A). Our high-resolution structure also allows a detailed conformation of TM helices, except for the M2 re-entrant loops that form the cation selectivity filter on the intracellular side. The core of the channel is formed by four M3 helices surrounded by peripheral M1 and M4 helices, which mediate the inter-subunit interactions as previously shown. The pore contains four residues—Met 664, Thr 660, Ala 656, and Thr 652—which form spatial constrictions on the extracellular side. The ion channel pore is especially narrow at Thr 660, with a pore radius of less than 1.15 Å, similar to that of the apo state GluA2 AMPAR (PDB ID: 4U2P). The overall pore formation is similar to that of the glutamate bound desensitized GluK2, except around Thr 652, where the pore is slightly wider than that in the desensitized state but still narrower than 1.4 Å (which is the radius of a water molecule). Overall, the channel was tightly closed (Fig. 3B, C).

As suggested by the previous molecular dynamics (MD) simulation and mutagenesis analysis on isolated GluK2 LBD^34,35^, GluK2 LBDs are conformationally flexible and can be form the closed bi-lobe conformation in the absence of agonists. In summary, our apo state GluK2 structure shows the ligand-free GluK2 LBD bi-lobes closure in the context of intact tetrameric receptors, unlike other iGluRs. The LBD bi-lobe closure indeed facilitates the dissociation of LBDs and thus promote the receptors forming desensitized-like four-fold conformation in detergent micelles.

### Activation of GluK2 KAR by partial agonist

KARs desensitize rapidly with saturated concentration of glutamate. Because GluK2 KARs desensitize within 10 ms, the previously determined structures of KARs in the presence of glutamate were considered to be in a desensitized state. In this study, we employed the naturally produced neurotoxin domoate, a highly potent KAR partial agonist, at both native and recombinant KARs^36–40^. To evaluate the efficacy of domoate, we first conducted whole-cell patch-clamp electrophysiological recordings from homomeric rat GluK2 WT expressed in HEK293T cells, and then compared the peak current amplitudes and desensitization kinetics activated by either glutamate or domoate (Fig. 1 A, B). Consistent with earlier observations^41^, the saturated concentration of domoate evoked current amplitude (10 μM) was approximately four times smaller than the glutamate evoked current (10 mM) at a holding potential of −70 mV, with mean current density of 250 ± 60 and 874± 80 pA/pF, respectively (Fig. 1A). The kinetic analysis revealed that the desensitization of domoate activated GluK2 was best fit by the sum of the fast and slow exponential functions (τ_desen-dom-fast_: 55.6 ± 4.70 ms, τ_desen-dom-slow_: 3134.8 ± 655.9 ms, *n* = 5), with the slow component being dominant (the quantification of percent desensitization for the slow component compared to the fast one was 61.9 ± 5.20%. We observed relatively “non-desensitizing” steady-state currents in the presence of the saturated concentration of domoate compared to glutamate evoked currents. Therefore, we hypothesized that domoate stabilizes receptors in a long-lasting, domoate bound intermediate state between the resting and activated/desensitized states, as seen in the patch-clamp recording, which is distinct from the glutamate bound fully desensitized conformation.

### Partial agonist bound KARs adopt multiple conformations

To gain insight into the conformational changes in GluK2 KAR induced by the binding of partial agonist domoate, we next determined the cryo-EM structure of domoate bound intact GluK2 KAR. The reported EC_50_ of domoate for GluK2 WT was 1.4 ± 0.25 μM^37^. Thus, we incubated the purified protein with a saturated concentration (5 mM) of domoate^42^ prior to grid freezing. The 3D classification revealed four classes of domoate bound GluK2 KAR complexes (Fig. S5, Table S2) with overall resolutions of 3.9–4.3 Å, as estimated by Fourier shell correlation (FSC) using the 0.143 cutoff without the application of symmetry (Fig. S3). The 3D reconstructions of all classes also showed a typical KAR assembly with the ATD, LBD, and TMD layers. The assembly of one of the major classes was stabilized in the desensitized conformation in the presence of domoate with the ion channel pore tightly closed, similar to the glutamate bound GluK2 structure. In contrast, the other three classes represented previously unseen domoate bound intermediate states with distinct LBD domain orientations and arrangements. The ion channels of these classes appeared to be closed with distinct pore features. Therefore, we named these four states domoate bound “desensitized,” “non-active 1,” “non-active 2,” and “non-active 3” with particle distributions of 30.9%, 12.6%, 22.3%, and 30.9%, respectively (Fig. S5). The local resolutions of the ATD layers of all four classes were higher than those of the LBD and TMD layers due to conformational stability (Fig. S3). The ATDs formed the classical clamshell-like conformation, and the subunit AB and CD pairs assembled dimers, as observed in the apo GluK2 structure. Comparison of ATD layers revealed that all classes contain the ATDs superposable to the unliganded apo state (Fig. S6A). However, when we superpose the TMDs and analyze the ATD position in term of the full-length structures, we observe that ATD swings by 12 Å compared to the unliganded apo state structure (Fig. S6B). Importantly, the ATD layers were at least 7.2 Å away from the LBD layers, similar to the previously reported structures^16,20^. Since there were no functional state-specific ATD–LBD interactions, regardless of the ATD swings, we concluded that the ATD movement is not critical for gating of KARs, unlike AMPARs^43^ and NMDARs^44–46^. Thus, in the next section, we will focus primarily on conformational changes in the LBD and TMD layers.

### Partial agonist binding-induced LBD domain closure and rearrangement in full-length GluK2.

We observed clear densities for domoate in the agonist binding pockets in all four classes, except subunit D in the minor class named “non-active 1” (Fig. S4, h). The ligand densities were accommodated within the agonist binding pocket between the D1 and D2 clefts in each LBD. Thus, we fitted the domoate models using the crystal structure (PDB ID: 1YAE)^32^ as a guide (Fig. S4e–g, i–l), although the precise ligand binding modes were not clearly resolved due to the resolution limitation. Previous structural studies have shown that domoate binding partially closes the LBD cleft (D1–D2 closure degree: 24–25°) compared to glutamate binding, which closes the cleft to a greater extent (D1–D2 closure degree: 33–36°)^22,32,47^. In our structures, domoate closed the LBD bi-lobes and stabilized similar conformations as the crystal structure of domoate bound Gluk2 LBD with an RMSD of 1.6–2.2 Å with the D1–D2 closure degree of 33–38°. Interestingly, we barely observed D1–D2 twisting motions in the domoate bound intact GluK2 structures, unlike previously suggested^48^. To identify how the domoate induced local rearrangement of LBDs changes the overall conformation of the intact tetrameric receptors, we next compared the assembly of the unliganded apo-and domoate bound intact GluK2 KAR structures.

**Figure 4.**
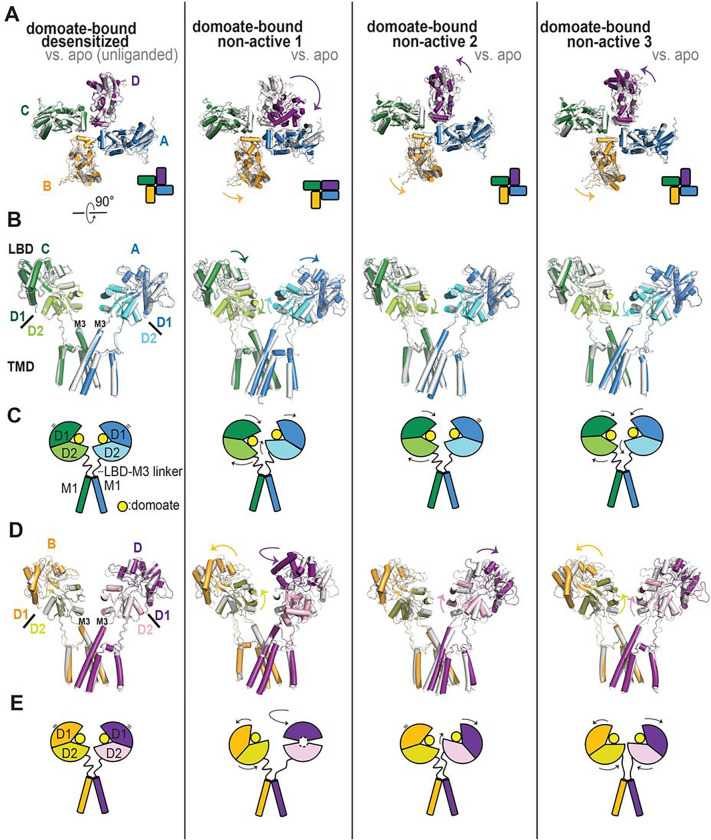
Conformational variability of domoate bound GluK2. Three-dimensional classes of the domoate bound GluK2 dubbed desensitized, non-active 1, non-active 2, and non-active 3. Subunits A, B, C, and D of domoate bound GluK2 are colored in cyan, yellow, green, and purple, respectively. Structures of domoate bound GluK2 are aligned to that of the unliganded apo GluK2 structure (gray) in (A), (B) and (D). **(A)** Extracellular view of the LBD layers for domoate bound GluK2 structures aligned to the unliganded apo GluK2 structure. Subunit arrangement at LBD is represented in cartoon. **(B, D)** Side view of the A–C or B–D pairs. Depicted are the conformational changes for the LBDs underlying the transition, as compared to the apo state. **(C, E)** Schematic presentation of the domoate bound conformational changes.

A comparison of the overall structures revealed multiple domain rearrangements that might be related to the gating of KARs. The domoate bound “desensitized” class, a major 3D class with approximately 30% population, showed an approximately four-fold rotational symmetry arrangement of tetrameric LBDs. While the LBDs of the A and C subunits in this class formed similar conformations with RMSD_A-C_ 0.58 Å, the LBD of the B and D subunits displayed more conformational variety (RMSD_A-B_ 1.45 Å, RMSD_B-C_ 1.58 Å, and RMSD_B-D_ 1.31 Å) with a small degree rotation of the D1 lobe compared to the apo state structure (Fig. 4B–E). The D2 lobes of the AC pair were superposable over the apo state. The top view of the LBD layer was nearly identical to the desensitized structures except for the small angle (2.5–6°) further in-plane rotations (Fig. 4A). Viewing the receptor from the side, we observed approximately 2° minor domain rotation perpendicular to membrane, and the minimal twist of D1–D2 lobes ranging 0.4–1.5 Å induced by the domoate binding (Fig. 4B–E). Overall, this domoate bound desensitized class displayed a superposable architecture over the unliganded apo desensitized structure, except for the ATD swings of 5.2 Å. Although not as strong as full agonists, our electrophysiology data showed that domoate cause rapid desensitization (GluK2 WT τ_desen_: 60.8 ± 9.1 ms) (Fig. 1B), indicating that this class represents a domoate bound desensitized conformation. The most minor class (“non-active 1”) populated 12.6% and resolved to 4.3 Å displayed an asymmetrical organization containing two LBDs forming a dimeric configuration, on one side, and a disrupted dimer (two separated monomer LBDs), on the other side (Fig. 4A), consistent with the previously reported asymmetrical structures of GluK3 complexed to the competitive antagonist UBP301 or UBP310 and GluK2 complexed to neuropilin and tolloid-like 2 (NETO2) auxiliary protein^17,18,49^. The LBD bi-lobe of subunit D, which forms a homodimer with subunit A, was stabilized in the partially closed-cleft conformation, similar to the domoate bound GluK2 LBD with an RMSD of 1.74 Å. As described above, at this resolution, we did not observe clear ligand density in the agonist binding pocket in subunit D, while the other three subunits showed a clear density of the ligands (Fig. S6 e-h). The structure of GluK3 in complex with the antagonists, which stabilized the LBDs in the open-cleft conformation, showed a similar asymmetrical conformation, suggesting that the degree of the D1–D2 cleft was not critical for the LBD dimer dissociation and receptor desensitization. Thus, how this asymmetric configuration is stabilized remains unclear. Compared to the unliganded apo-desensitized structure, the LBD of subunit D rotated by 110° anti-clockwise in the horizontal plane (Fig. 4A). Additionally, viewing from the side, we also observe LBDs of subunits A and B rolled up by 8° and 7°, respectively, while the LBD of subunit C rolled down by 8° (Fig. 4B, D). Overall, the AD pair of this class stabilized in a resting conformation, in contrast to the BC pair, which showed desensitized-like conformation. Thus, this class is considered an intermediate state between resting-active-desensitized and desensitized-resting states. The other minor class, named “non-active 2,” also displayed quasi-four-fold symmetrical LBDs with domoate molecules binding to all four subunits, similar to our domoate bound “desensitized” class. The top view of the LBD layer in this class showed less profound rotation for the B–D subunits by approximately 6°, while the A–C subunits were nearly identical to those in the unliganded apo desensitized state (Fig. 4A). While the AC pair showed minimal conformational changes (Fig. 4B, C), subunits B and D underwent different conformational changes perpendicular to the membrane and created asymmetrical conformation. Viewed perpendicularly to membrane, compared to the apo state structure, the LBD orientation in subunit D rotated up by 11° perpendicular to the membrane, while the D1 domain of subunit B showed very minor conformational changes with a 4° rotating down motion (Fig. 4D, E). Thus, we consider this is an intermediate state between the domoate bound “non-active1” and “desensitized” states. The last class (populated 30%), named “non-active 3,” also showed approximately four-fold symmetry but with distinct LBD rotation and orientation compared to the desensitized structures. All four LBDs underwent anti-clockwise rotation by 2–15° further than those in the unliganded apo desensitized structure (Fig. 4A). The domoate binding rotated down the LBDs in the AC pair slightly and rotated up the LBDs in the BD pair by 8°; therefore, the D2 lobes moved away from the membrane (Fig. 4B–E). As a result, these distinct AC and BD pair conformational changes generated unique subunit arrangements. The ion channel in this state is most tightly closed, similar to those in the “desensitized” conformation, as described below. This class may represent an intermediate state between the domoate bound “non-active 2” and “desensitized” states, or could be another conformation of a desensitized state, considering the LBD D2 orientation of the BD pair and the ion channel pore formation. Overall, this observation revealed that all four classes demonstrate distinct conformations of LBD layers.

### Desensitization ring controls the gating of KARs.

**Figure 5.**
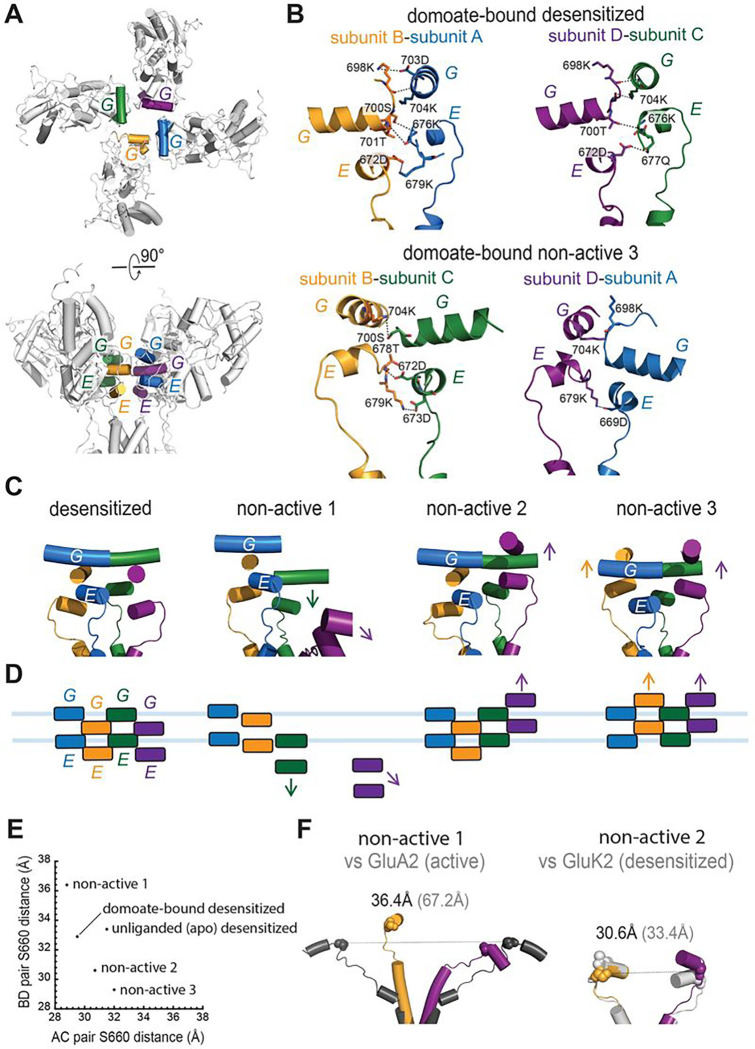
Desensitization ring in GluK2. **(A)** Top and side views of the LBD layer in the domoate bound desensitized state structure highlighting the desensitization ring E/G helices of subunits A–D colored in cyan, yellow, green, and purple, respectively. **(B)** Inter-subunit electrostatic interactions between G and E helices in the domoate bound desensitized and non-active 3 structures. **(C)** Desensitization ring formation in domoate bound GluK2 structures. Depicted are conformational changes for LBDs (and E/G helices) underlying the transition. **(D)** Schematic presentation of the desensitization rings in domoate bound GluK2 structures. The light blue bars represent the position of the E/G helices in the domoate bound desensitized structure. **(E)** Plot of the A–C versus the B–D distances measured between Cα of Ser660, comparing apo, domoate bound desensitized and non-active 1–3 structures. **(F)** Side views of the LBD-M3 linkers comparing non-active 1 versus activated GluA2 (PDB ID: 5WEO) (left) and non-active 2 versus glutamate bound desensitized GluK2 (PDB ID: 5KUF) structures. Tension of the LBD-M3 linkers measured by the distance between the two Ala671 Cα (spheres) is the major determinant for the opening or closing of the channel gate.

To evaluate the functional states of these structures, we further analyzed the local rearrangements of LBD and the linkers that connect the bottom (D2) lobes of LMDs and TM3 helices (“LBD-M3 linker”). According to previous studies, KARs have a unique ring-like motif, named “desensitization ring,” which defines their desensitization^16,19^. The two-layered ring is comprised G and E helices, which formed the top and bottom rings, respectively (Fig. 5A). In the glutamate bound desensitized GluK2 structure, the ring was mediated by staggered helix contacts between adjacent subunits stabilized by numerous hydrogen bonds and salt bridges, which has not been observed in other iGluRs to date^16^. Interestingly, our domoate bound GluK2 structure showed distinct arrangement of the desensitization rings, indicating that they represent different intermediate states. In some classes we also observed number of electrostatic interactions between helices which stabilize the “desensitization ring” formation (Fig. 5B).

The structure of the domoate bound “desensitized” state generated a two-fold symmetrical desensitization ring with alternating staggered helices viewed from the side (Fig. 5C, D). As expected, the desensitization ring in the domoate bound desensitized structure was nearly identical to those of our unliganded desensitized state structure and the glutamate bound desensitized structure with conserved interactions. The LBD-M3 linkers, connected to the E helices, were not well resolved due to their flexibility. Therefore, we analyzed the tension of the LBD-M3 linkers in the subunit AC and BD pairs measured by the distance between two Ser670 units to determine whether the ion channel gate was open or closed. As Figure 5E shows, the distance between the S660 residues in the AC and BD subunits of this desensitized conformation is very close to that in the apo desensitized structure. The relaxed LBD-M3 linkers, represented by the distance that coupled the TMD channel opening/closure, indicate that the ion channel remained tightly closed.

As described above, the “non-active 1” structure exhibits an asymmetric LBD arrangement with one dimer and two monomers. Due to the LBD dimer formation of the A and D subunits, this class contained a disrupted desensitization ring in the tetrameric receptors (Fig. 5C, D). Additionally, the LBD rigid-body rotating up motion of subunits A and B and the rotating down motion of subunit C rearranged the EG helices, which resulted in no inter-subunit contacts, in contrast to the extensive interactions in the desensitized structures. The LBD dimer formation separated the bottom of the D2 lobes in the BD pair. The distance between two S670 units was 36.4 Å (Fig. 5E), which is approximately 3.8 Å wider than the desensitized structure but not as wide as the competitive antagonist-bound GluK2, which contains LBDs forming dimer-of-dimers (PDB ID: 5KUH). In addition, we did not observe parallel movement to the membrane of these linkers, which resulted in the channel gate opening previously observed in the structures of activated AMPARs (PDB ID: 5WEO)^1,50,51^ and NMDARs (PDB ID: 6WHT)^52^. While the asymmetric LBD in the “non-active 1” conformation induced the mild tension in the LBD-M3 linkers of the BD, subunit, the linkers are not extended as the those observed in the activated GluA2 structure (Fig. 5F), indicating that the ion channel remained closed.

The minor LBD rotating down motion of subunit C and the major LBD rotating up motion of subunit D by approximately 10° in the “non-active 2” structure generated a unique asymmetrical desensitization ring (Fig. 5C, D). The G helices of the AC subunit moved 2 Å closer to each other, while those of the BD subunit moved away by 4 Å. Therefore, the desensitization ring formed in this “non-active 2” conformation was geometrically similar to the compact ring formed in the glutamate bound desensitized heteromeric GluK2/GluK5 than that of the desensitized homomeric GluK2 KARs^19^. A close inspection revealed that no interaction was formed between subunits AB and BC due to the movement of subunit B. The distance between S660 is shorter than the desensitized states and linkers show similar conformation as the desensitized states (Fig. 5E, F).

While the “non-active 1” and “non-active 2” states presented disrupted and unsymmetrical desensitization rings with minimal or no inter-molecular interactions, the desensitization ring in the “non-active 3” conformation displayed two-fold symmetry but a distinct staggered ring structure. The rotating down motion of the AC pair and the approximately 10° rotating up motion of the BD pair rearranged the straggling pattern of the desensitization ring with BD pair sitting on the top of the AC pair (Fig. 5D). Interestingly, the ring formation was stabilized by numerous inter-subunit interactions with many electrostatic interactions, which were observed in the desensitized structures (Fig. 5B). Therefore, the two-fold symmetrical ring formation in this class was more stable than the other two non-active conformations, which represented a distinct configuration of the domoate bound desensitization state or a transition state close to the fully desensitized receptors. The LBD-M3 linkers in the AC pair in the “non-active 3” conformation is even more relaxed than those in the apo unliganded-desensitized state due to the D2 lobe rolling down motion (Fig. 4), while the rotating up motion of the subunit BD pair pulled the LBD-M3 linkers slightly and generated linkers that were further extended perpendicularly to the membrane. However, these linkers did not show any movement parallel to the membrane. Overall, the LBD-M3 linkers in this “non-active 3” class remained relaxed and insufficient to open the ion channel.

### Ion channel in domoate bound GluK2.

**Figure 6.**
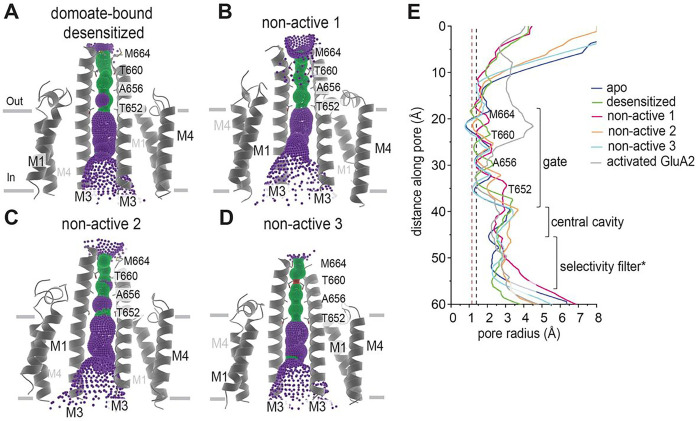
Ion channel structure of domoate bound GluK2 KARs. **(A–D)** Pore profiles of the receptors in different conformations of the domoate bound GluK2 KARs with two M3 helices and their pore-lining residues. The pore radius was calculated using HOLE, with regions having a pore radius less than 1.15 Å colored red, water-accessible sections with radius larger than 1.15 Å colored green, and sections with a radius larger than 2.5 Å colored purple. (E) Pore diameter comparison between the unliganded apo- and domoate bound GluK2 KARs and the activated GluA2 complex bound to glutamate, cyclothiazide, and stargazin (PDB ID: 5WEO).

The local resolutions of the TMDs are lower than those of the extracellular domains (Fig. S3). Nevertheless, our cryo-EM reconstructions showed a clear TMD density (Fig. S7) and facilitated the analysis of the pore structures. We observed that, in the TMDs, ion channel pores were formed by three transmembrane helices, M1, M3, and M4, consistent with the previously reported structures of the iGluR superfamily (Fig. 3 and Fig. 6)^1^. The M3 helices line the ion permeation pathway at the extracellular side and function as an activation gate, and the M1 and M4 helices surround the M3 helices and face the membrane lipids. In our structures, the small re-entrant helix (M2) helices, which line the intracellular side of the pore^20^, were not resolved due to conformational flexibility. We observed the densities of the side chains of the conserved SYTANLAAF motif and M1–M2 loops, called the cap domain, on the intracellular site of the ion channel entrance, as previously reported^20^ (Fig. S7).

A comparison of these TMDs revealed ion channel pore formation. In the domoate bound “desensitized” state, the ion channel was tightly sealed at the bundle crossing of the M3 helices Thr652, Ala656, and Thr660 (Fig. 6A), although the pore radius at residue Thr660 was not as narrow as those in the unliganded (apo) and glutamate bound desensitized GluK2 KARs. Considering the extracellular domain formation and this central pore radius, we concluded that this class is a domoate bound desensitized state stabilized in a structure similar to the glutamate bound desensitized conformation. The central pore of “non-active 1” was the widest in all the domoate bound GluK2 structures. The LBD dimer generated a disrupted desensitization ring, and the LBD-M3 linker orientation created an asymmetrical pore, expanding the ion channel pathway by 0.2 Å compared to the desensitized states. However, the pore radius at Ala656 and Thr660 were smaller than 1.4 Å, which is the radius of the water molecule, indicating that the ion channel of this state remains closed (Fig. 6B). The central pore in the “non-active 2” conformation is between the “non-active 1” and “desensitized” states; therefore, the ion channel remained closed (Fig. 6C). Finally, the ion channel in “non-active 3” was tightly sealed at the bundle crossing of the M3 helices Thr652, Ala656, Thr660, and Met664, with the narrowest point at Thr660 (Fig. 6D) and showed a very similar ion channel path to the desensitized conformations. Considering the rearrangement of the LBD and symmetrical desensitization ring, this class could possibly represent another desensitization state distinct from the glutamate bound desensitized state.

### N-glycans mediate the ATD–LBD intra-and inter-subunit interactions and regulate the gating of the partial agonist-activated GluK2 through the anion and cation binding sites.

**Figure 7.**
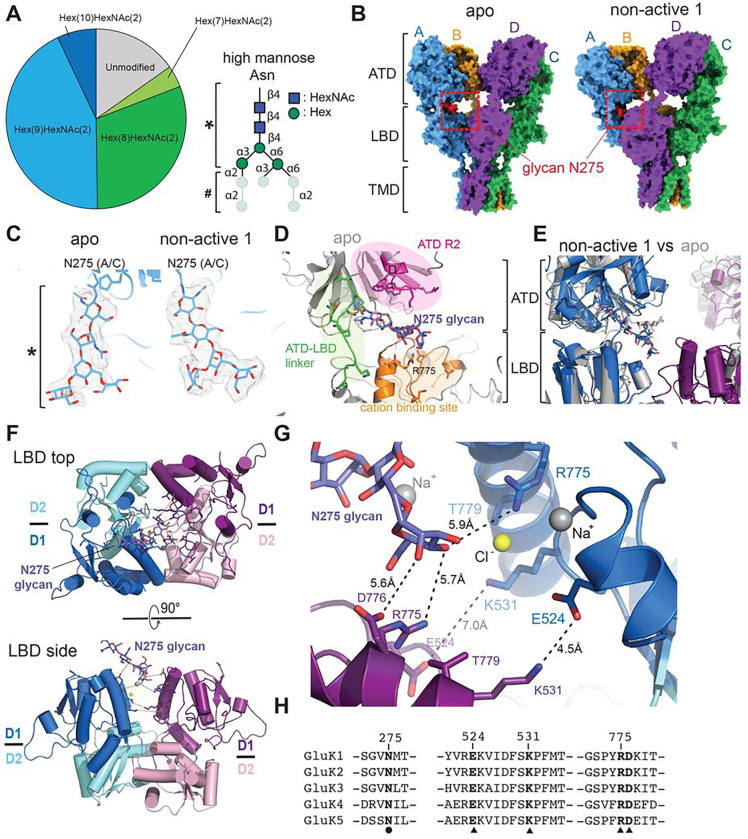
*N*-Glycans on ATD interacts with the anion and cation binding sites at the LBD dimer interface. **(A)** Left: Quantification of *N*-glycan on the purified GluK2 KARs using LC-MS/MS. Four distinct forms of glycosylation were detected. Right: Schematic of a high mannose (* and # are regions showed a clear or invisible EM density in our 3D reconstitutions). **(B)** EM density maps of *N*-glycans at Asn275 in ATD of the apo and non-active 1 conformation. *N*-glycans are colored red. In the unliganded apo state structure, LBDs formed an approximate four-fold symmetrical conformation, and the N275 glycan on ATD formed inter-domain, intra-molecule interactions with LBD. In the domoate bound non-active 1 structure, LBD dimerization of the subunit AD pair promoted the N275 glycan forming inter-domain interactions with the LBD of both the A and D subunits. **(C)** Cryo-EM density for resolved Asn275 glycans in the apo and “non-active 1” structures. **(D)** The N275 glycan in the apo state structure. The glycan is surrounded by the ATD R2 domain, ATD-LBD linker, and LBD D1 lobes, which limit the space, and stabilize the N275 glycan located at the top of the anion and cation binding sites. **(E)** Structural alignment of the apo and non-active 1 structures and the location of the N275 glycan. **(F)** Top and side view of the LBD dimer with the N275 glycan on top of the LBD dimer interface in the “non-active 1” conformation. The predicted chloride and sodium ion positions are shown by superposing the crystal structure of Na^+^- and Cl^−^- bound GluK2 LBD in complex with glutamate (PDB ID: 2XXR). **(G)** N275 glycan and Na^+^ and Cl^−^ binding sites at the LBD D1-D1 interface between the A and D subunits. The positions of the ions are predicted by superposing the crystal structure. The dashed line indicates the electrostatic interactions between the N275 glycan and residues forming the Na^+^ and Cl^−^ binding sites located on the LBD D1–D1 domains in the subunit A and D. **(H)** Amino acid sequence alignment and the position of the N275 glycan. (●) is the conserved N275 residue, and (▲) are the conserved residues contributing the anion, and cation binding in GluK1-GluK5 KARs.

The sequence analysis and previous studies have revealed that rat GluK2 KAR has nine *N*-linked glycosylation sites^53^. Previous structures of intact KARs have also shown EM densities corresponding to the carbohydrates of *N*-linked glycosylation on the ATD and LBD of KARs^16,18,19,49^. In addition, electrophysiology data have shown that these glycans affect the functions of receptors at the levels of multisubunit assembly, protein trafficking, ligand binding, and the gating properties of receptors^17^. To identify the *N*-glycan population and composition of the purified GluK2 KAR used for the structural studies, we first expressed the intact GluK2 in *Sf9* insect cells and characterized the *N*-glycosylation. The purified GluK2 EM protein sample was digested with trypsin and chymotrypsin, analyzed using capillary column liquid chromatography with tandem mass spectroscopy (LC-MS/MS), and the types of glycosylation were determined using the Sequest program. The results indicate that there are nine *N*-glycosylation sites at Asn 67, 73, 275, 378, 412, 423, 430, 546, and 751 with various glycan forms. The LC-MS/MS result indicated that the *N*-linked glycosylation on GluK2 can have three to seventeen forms depending on the position of glycosylated Asn. Four different forms of *N*-glycans at Asn275 were identified with two major types of glycans: 8 hexoses and 2 N-acetylhexosamines [Hex(8)HexNAc(2)] and 9 hexoses and 2 N-acetylhexosamines [Hex(9)HexNAc(2)] (Fig. 7A). In the EM maps, we observed residual EM densities of four N-linked glycosylation near Asn275, Asn378, and Asn412 on ATD, and Asn430 on LBD of the AC subunits, while the BD subunits showed only two evident glycan densities near Asn378 and Asn412. We specifically focus on the two N-glycans at Asn275 and Asn430 formed inter-domain glycan–protein interactions between the ATD and LBD layers.

Our EM maps allows clear assignment of two N-acetylhexosamines (HexNAc) and three hexoses (Hex) of the Asn275 *N*-glycan at the ATD–LBD interface in both apo and “non-active 1” conformations (Fig. 7B, C), similar to the GluK2 and GluK3 maps solved at 3.8 and 7.4 Å, respectively^16,17^. The rest of the Hex (Fig. 7A) were not visible due to the flexibility of glycans. The N275 glycan was surrounded by numerous polar and bulky residues, which positioned the glycan between helices D in the lower domain (R2) of ATD, the ATD–LBD linker, and the upper domain of LBD (D1) (Fig. 7D). A comparison of the positions of the N275 glycan in the apo and “non-active 1” states revealed that this glycan was positioned similarly in both structures (Fig. 7E). As described above, the subunit AD pair in the “non-active 1” conformation contained an approximately two-fold symmetric LBD dimer. Interestingly, the N275 glycan was located at the center of the LBD dimer and interacted with both protomers two-fold symmetrically and mediated the inter-subunit and inter-domain interactions. In particular, spatial restriction promoted the N275 glycan sitting on the anion and cation binding sites that control KAR desensitization (Fig. 7F).

The LBD dimer of the subunit AD pair in the “non-active 1” conformation formed similar to the previously solved crystal structure of the isolated GluK2 LBD dimer complexed to glutamate (PDB 2XXR). However, we also observed some differences, such as the distance between the LBD D1-D1 interface and the D1-D1 contacts, which affected the cation binding on LBD. A closer observation revealed that the two-fold inter-subunit polar interactions between Glu524 and Lys531 were the major interactions that stabilized the LBD dimer formation (Fig. 7G). The E524 and K531 side chains formed water-mediated ionic interactions with a distance of 14.1 Å between the Cα of the E524 and K531 residues. This is approximately 3.8 Å wider than the crystal structure, which shows E524–K531 Cα distances of 10.8 and 11 Å. Additionally, the distance between the side chains of two Arg775 residues in AD subunits in the “non-active 1” class was approximately 9.8 Å (Cα-Cα distance: 20.7 Å), which is approximately 7.2 Å wider than the crystal structure, which shows the R775-R775 side chain distance of 3.3 Å (Cα-Cα distance: 13.5 Å). Importantly, in our “non-active 1” conformation, the N275 glycan occupies the space between these two R775 residues and formed two-fold symmetric, glycan-mediated inter-protomer interactions (Fig. 7F, G). The R775 side chains and the carbohydrate of the N275 glycan form water-mediated hydrogen bond networks, which stabilize LBD dimer formation in addition to the E524–K531 interactions. We did not observe any EM densities of ions and water molecules due to the resolution limitation, but the structural alignment with the crystal structure revealed that the N275 glycan locates within 3–5 Å distance from the chloride binding site^35^. Thus, we assumed that the N275 glycan stabilizes the position of R775-charged side chains, directly interacts with the chloride ion, and electrostatically stabilizes an anion within the anion binding pocket. The distances between the N275 glycan and the Cα of Lys531, the residue that also consists of the anion binding sites in each dimer, were 9.9 Å and 10.8 Å, and we did not observe any direct interactions.

Furthermore, this N275 glycan was positioned close to the sodium binding sites with 7.8–8.7 Å (Asp 528 Cα) and 10.4–10.7 Å (Glu 524 Cα). According to our MS analysis, the major glycan compositions were 8 and 9 Hex and [Hex(8)HexNAc(2)], respectively (there were 6 additional mannoses, which we did not observe in the EM densities due to conformational flexibility). Thus, we assumed that the N275 glycan also stabilized the sodium binding pocket formed by E524 and D528. Finally, the carbohydrate of the N275 glycan also formed water-mediated ionic interactions asymmetrically with a 5.6 Å distance between D776 and N275 glycan (glycan-D776 Cα:−8.0 Å), perhaps electrostatically stabilizing the sodium binding site. This structural information indicates how the N275 glycan supports dimer formation, stabilizing the sodium and chloride ion binding sites. In particular, the sequence alignment shows that all of these N275 glycan sites and glycan N275 interacting residues, E524, K531, and R775, are fully conserved in GluK1–GluK5 KARs (Fig. 7H) but only partially conserved in AMPARs. Therefore, it is presumably the unique regulation mechanism of KAR activation and desensitization by glycans.

In addition to the N275 glycan, we observed two N-acetyl glucosamines (or HexNAc) of the oligo mannose core of the Asn 430 *N*-glycan, located on the LBD D1 domain and mediating the inter-domain and intra-subunit interactions with the ATD layer. The second HexNAc of Asn 430 glycan forms CH-π interaction with the aromatic ring of Tyr 274 located on the lower lobe (R2) of ATD, observed only in the AC subunits due to the two-fold symmetrical arrangement. This ATD–LBD interaction through the Asn 430 glycan is observed in all apo-and domoate bound GluK2 in this study, as well as the previously determined GluK3 structure^17^. The electrophysiological data show that the N430 glycan knockout (KO) or mutating Y274F on GluK3 mildly slows down the desensitization and increases the recovery from the desensitized state. Considering that the LBD of the AC subunits undergo relatively small movements, in contrast to the large rotation of the BD subunits upon activation and desensitization, this glycan–protein interaction mainly contributes to the receptor assembly functional state independently.

### Asn 275 glycans interact with the anion and cation binding sites on LBD and modify deactivation and desensitization.

The “non-active 1” structure showed that the N275 glycan interferes with the sodium and chloride binding sites at the LBD D1-D1 dimer interface. Therefore, we hypothesized that the N275 glycan KO or R775A mutation destabilizes the anion and cation binding sites, and therefore, the LBD dimer formation, which modulates the KAR gating. To validate our hypothesis, we generated three mutants (T277A, R775A, and T277A/R775A), knocking out the N275 glycan and/or R775, and analyzed the gating properties including deactivation and desensitization microscopic kinetics of GluK2 KAR activated by either glutamate or domoate, as well as receptor recovery from desensitized state using whole-cell patch clamp electrophysiology. The T277A mutation knocked out the *N*-glycan at Asn275 by mutating Threonine 277 to Alanine to modify the consensus *N*-glycosylation sequence (N-X-S/T where X≠ P).

**Figure 8.**
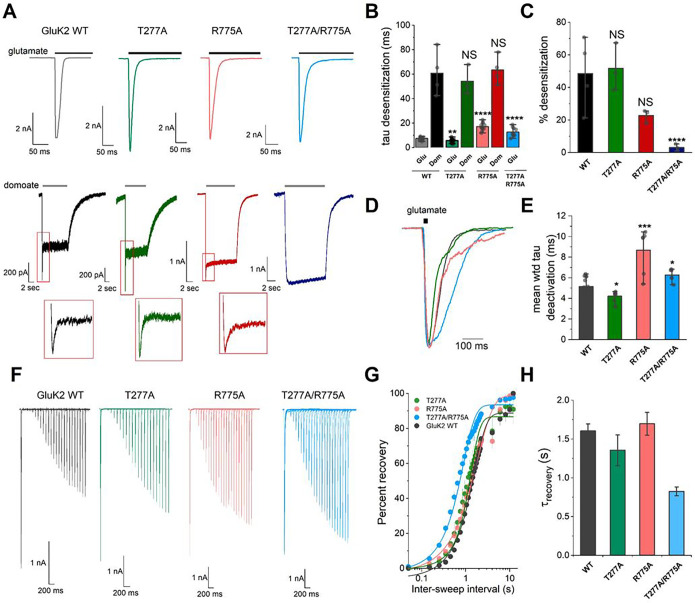
Effect of N-linked glycosylation on KAR gating properties. **(A)** Representative whole-cell currents evoked by a 1 s of glutamate (10 mM, black bar) or 10 s of domoate (10 μM, gray bar) from recombinant GluK2 WT or mutant receptors expressed in HEK293T cells. The holding potential was −70 mV in all recordings. The responses to domoate showed two exponential curves. Inset: exponential fit of the early onset desensitization time course. **(B)** Quantification of the desensitization fitted the current decay using a single exponential function and calculated the tau desensitization. Summary bar graph of desensitization of receptors activated by glutamate (light colors) or domoate (dark colors) showing the different desensitization kinetics for WT and mutant receptors. **(C)** Quantitation of percent of desensitization for domoate evoked currents comparing the mean amplitude of the earlier and plateau-like components**. (D)** Representative normalized current traces for GluK2 WT and mutant receptors. The deactivation time constants were measured for each mutant activated by 10 mM of glutamate for 3 ms. **(E)** Quantitation of the deactivation tau values fitted to either a single exponential component, for WT and the T277A mutant, or two exponential components, for R775A and T277A/R775A mutant receptors. **(F)** Representative traces obtained from the application of a two-pulse protocol from 50 ms to 12 s with Δ100 ms for generation of the time course of recovery from desensitization. 10 mM of glutamate was applied for 50 ms. **(G, H)** Quantitation of recovery from desensitization. The tau recovery was obtained from the fit-to-one exponential function. In bar graphs, each point represents an independent measurement, and the error bar represents SEM. The evaluation of significant differences was performed by comparing each mutant against GluK2 WT. All graphs drew from two to four separate experimental days. Statistical significance was determined using a two-sample unpaired t-test and is denoted as *p < 0.05; ** p < 0.01; *** p < 0.001; **** p < 0.0001, **** P < 0.0001. NS, Not significant, WT, Wild type; wtd, weighted.

First, we analyzed the desensitization microscopic kinetics of rat GluK2 activated by glutamate. The N275 glycan KO (T277A) mutation promotes the receptor desensitization, similar effects of KAR glycans reported earlier^17,54^ (Fig. 8A, B, Table S3). In contrast, we observed that the R775A single mutation, which is known to be more favorable to stabilize the LBD dimer, slower the desensitization as previously proposed^35^. Earlier structural studies have shown that a lack of visible chloride binding on the chloride site is the only structural difference in the R775A-mutated GluK2 LBD crystal structure compared to the WT structure^35^. Therefore, we further generated the construct containing two mutations, T277A/R775A and analyzed the effect of N275 glycan. A comparison of the R775A and T277A/R775A mutants revealed that T277A perhaps changes the charge balance at the LBD interface in the GluK2 R775A backbone construct and speeds the desensitization compared to the GluK2 R775A single mutant yet is desensitized slower than GluK2 WT (Fig. 8A, B). Percentage desensitization of GluK2 mutants to domoate are distinct from the GluK2 WT, specifically mutants containing R775A mutation show less desensitization (Fig. 8C, Table S3). The T277A and R775A single mutants evoked by domoate show similar tendencies as the glutamate evoked GluK2 mutants. However, the T277A/R775A double mutant does not display a rapid component of desensitization indicated that the effect of the N275 glycan in the desensitization of GluK2 activated by full- and partial agonists are similar but not identical.

Next, we analyzed the deactivation microscopic kinetics evoked by glutamate. The T277A mutant showed slightly faster deactivation, in contrast to the slower deactivation kinetics of R775A. Similar to the effect of N275 glycan on receptor desensitization, the T277A/R775A double mutant displays the faster deactivation than the R775A single mutant (Fig. 8 D, E, Table S3).

Furthermore, we analyzed the rate of recovery from desensitization by generating recovery currents via two applications of glutamate at different time intervals. We observed that the T277A mutant recovers faster than WT or R775A mutant, which agrees with the previous observation on GluK3^17^ (Fig. 8F–H, Table S3).

R775A demonstrates slower recovery, indicating the key role of N275 glycan and the residue R775 in receptor recovery from desensitization. Similar to the comparison of the WT and the T277A mutant, the T277A/R775A shows slower recovery than the R775A mutant. In summary, these structural and electrophysiological data explain how the N275 glycan interface with the anion and cation binding sites at the LBD D1-D1 interface and modifies the deactivation, desensitization, and recovery from desensitization of homomeric GluK2 KARs.

## Discussion

All of the previously determined intact KAR structures represent either inactivated or desensitized states. Therefore, the detailed KAR gating mechanism has remained elusive. Our current work provides the cryo-EM structure of GluK2 in the absence of ligands (apo) and in complex with the partial agonist domoate. Our major findings are that (1) unliganded apo GluK2 forms a desensitized-like conformation; (2) domoate destabilizes the “desensitization ring” formation and attenuates the desensitization; and (3) the *N*-glycan at Asn275 on ATD interferes with the cation and anion binding sites located at the LBD dimer interface and modulates KAR gating.

### KAR LBD bi-lobe closure in the absence of agonists desensitize receptors without prior activation.

First, we observed that the unliganded apo GluK2 conformation resembles the desensitized states. The first high-resolution apo-state structure of KARs, reported here and solved at 3.0 Å, was reconstructed and refined with C1-symmetry. In this structure, the LBDs of all four subunits were stabilized in closed LBD bi-lobe conformations with variable bi-lobe closure degrees, similar to either the full or partial agonist-bound fully or partially closed LBD conformations, which are not as open as the apo-state conformation of AMPAR and NMDAR LBDs. Unlike the energetically favorable LBD bi-lobe open conformations in the apo AMPARs and some apo NMDARs^44,55–58^, earlier computational studies showed that isolated apo GluK2 LBD exhibited the extremely high flexibility^34^. Our structural study demonstrates that GluK2 LBDs preferably form a closed conformation in the context of the intact tetrameric KARs in detergent micelles. Consequently, the LBD bi-lobe closure decouples LBD dimers in the full-length receptor and populates four-fold symmetric conformations. All our three-dimensional (3D) reconstructions of apo-GluK2, including two low-resolution classes, showed similar four-fold symmetric conformations, indicating that this is the major conformation under the given condition. Our apo GluK2 structure is indeed superimposable on the structure of the glutamate bound GluK2 in a stable desensitized state, except for the minor differences in the TMD layer, where the ion channel contains three constrictions with the narrowest point at Thr660, while the channel in the glutamate bound desensitized structure has two narrow, water inaccessible constrictions at Thr652 and Thr660. Nevertheless, the apo structure features a closed ion channel.

The single-molecule fluorescence resonance energy transfer (smFRET) analysis revealed that apo KARs exist in both classical two-fold symmetric resting-like conformations and four-fold desensitized-like conformations in the absence of agonists^59,60^. Additionally, the structures of apo homomeric GluK2 (determined by cryo-electron tomography at 21 Å) and apo heteromeric GluK2/GluK5 KARs (determined by cryo-EM at 7.5 Å) showed the two-fold symmetric conformation of LBD layers^19,33^. The 3D reconstruction of GluK3 analyzed by negative stain data (resolution not described) also showed the presence of LBD dimers^18^. However, these relatively low-resolution data suggested a high level of conformational heterogeneity and flexibility of KARs, indicating the co-existence of multiple conformations of apo-state KARs.

Notably, the receptor desensitization prior exposure to agonists has previously been observed in other excitatory receptors, including AMPARs^23–26^. Similarly, it has been shown that GluK2 KARs also microscopically desensitized with or without prior channel activation^61^. Additionally, earlier studies showed that the LBD dimer decoupling initiated KAR desensitization^35,62^. Therefore, we concluded that we captured receptors in an unliganded constitutive desensitized state consistent with the expectation from electrophysiological and MD simulation analysis. Our apo state GluK2 structure reveals that the desensitized state of GluK2 KARs can be accessed from both resting and active states, and that the desensitized conformation with four-fold symmetry is presumably more energetically stable in the absence of agonists, unlike other iGluR subfamilies. Further electrophysiological studies will be required to confirm this high level of flexibility of KAR LBDs increases the proportion of KARs in the unliganded constitutive desensitized states.

### Intermediate structures revealed a KAR-specific mechanism for how the partial agonist destabilizes the desensitization ring and attenuates desensitization.

The most important feature of the partial agonist domoate is that it exhibits a non-desensitizing current with a significantly smaller amplitude at the saturated concentration compared to full agonists. We obtained four domoate bound intact GluK2 structures with distinct tetrameric arrangement of LBD layers with a similar LBD bi-lobe closure degree as the crystal structure of domoate bound GluK2 LBD^32^. While previous reports have suggested that the D2 domain twisting motion relates to non-NMDAR partial agonist activation and desensitization^35,48,63^, here, we barely observe an LBD D1-D2 twisting motion (<1°). Therefore, we conclude that LBD twisting is not the most critical factor for control receptor desensitization. Instead, our structural results suggest the destabilization of the desensitization ring formation may block receptor desensitization. The desensitization ring comprised of helices E and G located on the lower (D2) lobe of the LBD was proposed previously to control KAR gating^16,19^.

Our unliganded or domoate bound desensitized states show a two-fold symmetrical desensitization ring in the four-fold symmetric intact tetrameric receptor with numerous inter-subunit polar interactions, which stabilize the ring formation similar to the that of the glutamate bound desensitized state conformation. Previous studies have shown that disrupting the inter-subunit interactions between G helices speeds up recovery from desensitization^16^, indicating the importance of the stabilization of this ring formation. In contrast, the domoate bound non-active conformations show either disrupted (non-active 1), asymmetrical (non-active 2), or symmetrical but distinct staggered (non-active 3) pattern of desensitization rings. The inter-subunit polar interactions observed in the desensitized states are no longer formed in those intermediate classes.

The ion channels in these intermediate state structures are slightly wider than those in the desensitized structures. However, the LBD-M3 linkers, which control channel opening, remain relaxed compared to those in the activated GluA2 AMPAR structure, and therefore, ion channels in domoate bound conformations are closed. In conclusion, we observed that the domoate binding on receptors facilitate GluK2 forming asymmetrical or disrupted desensitization ring formation with no inter-molecule interactions, which attenuates the receptors’ desensitization.

### N-Glycosylation at Asn275 interacts with anion and cation binding and regulates KAR gating.

While multiple studies have shown that *N*-linked glycosylation modulates the localization and functional properties of KARs^17,54^, the detailed molecular mechanism was unknown up to now. Our LC-MS/MS analysis of *N*-glycans showed that there are nine glycosylation sites in GluK2 with various glycan forms. Importantly, our structures show the detailed glycan–protein interactions mediated by glycans at Asn275 and Asn430 at the ATD–LBD domain interface. We observe a well-resolved electron density of *N*-glycan at Asn275 located at the top of the cation and anion binding sites at the LBD dimer interface in the non-active 1 conformation. Earlier structural studies on an isolated LBD dimer have proposed that chloride binding stabilizes sodium binding at the LBD dimer interface, and hence, it stabilizes dimer assembly^35,64^. However, in our tetrameric GluK2 structure, two protomers are not as close as the crystal structures. For example, the distance between two R775 residues, which serve as the chloride binding site, is approximately 7.2 Å wider than those observed in the crystal structure of the GluK2 LBD dimer^35^. In the crystal structure, two R775 side chains directly formed a salt-bridge across the LBD dimer. Instead, we observe that the N275 glycan interact with side chains of R775 of both protomers two-fold symmetrically and mediate the inter-subunit interactions. Although we do not observe sodium ions and the rest of the mannose due to resolution limitations and the flexibility of glycans, analysis of sodium ion binding using the crystal structure as a reference indicates that this N275 glycan locates close to the Na^+^ ion and affect to the electrostatics around the cation binding site in addition to forming interactions with residues which shape the sodium binding site. Our patch-clamp electrophysiological recordings show that the N275 knockout mutation destabilized the LBD dimers and modified deactivation, desensitization, and recovery from desensitization.

In addition, our high-resolution structure of apo GluK2 KAR show that N430 glycans in subunits A and C form intra-molecule CH-π interactions with Try274. All of the domoate bound GluK2 structures show that the AC subunits undergo minimal conformational changes, in contrast to the drastic movement of BD subunits upon desensitization, indicating that this N430 glycan stabilizes the assembly of the GluK2 functional state independently, and therefore, the Asn275 glycan is the major *N*-glycosylation that modulates the receptor gating.

In summary, this structural study concluded that apo GluK2 KAR, which contains the unique highly flexible LBDs, can form an unliganded desensitized state. Based on the current structural data, we propose that the proportion of unliganded desensitized states of KARs is greater than the proportions of AMPARs and NMDARs. Our structural results suggest that domoate destabilizes the desensitization ring and attenuates KAR desensitization. While the activation mechanism of KAR remains elusive, this study illuminates the conformation alternation and non-desensitizing mechanism of GluK2 KAR by domoate. We also demonstrate that the N275 glycan in the AC subunits may directly interact with the Na^+^ and Cl^−^ ions as well as residues forming the cation and anion binding sites, and modulate KAR gating kinetics. Considering the distinct structural properties of non-KAR iGluRs, we conclude that the mechanism we report here is unique in KARs. Important questions that remain unanswered include how the conformational transitions of KARs upon activation by full- and partial agonists are distinct and how other structurally related or unrelated partial agonists control receptor functions distinctively. These glutamate analogs including kainate and domoate are known to be neurodevelopmental toxins, but conversely, they are also considered as potential drugs to treat brain diseases including epilepsy. Therefore, fully understanding the kinetics as well as neurotoxicity and cellular mechanisms is valuable to further elucidate KAR functions in a physiological or pathophysiological context.

## Materials and Methods

### Plasmid construction

The construct used for the structural studies was full-length rat GluK2 KAR (GenBank 54257, Uniprot code P42260). The GluK2 gene was RNA-edited at position 567 (I to V) and had two mutations of C576V and C595S, which promote protein expression^16,33^. The gene was cloned into the pFp10_hsp vector harboring sequences for the hr1 enhancer, the Drosophila melanogaster HSP70 promoter, and the p10 3’ UTR, which has been established previously^30,52^, and combined with a human rhinovirus 3C protease recognition site and a Twin-Strep affinity tag at the C-terminus. The construct was referred to as GluK2 EM.

### Cell culture

#### Mammalian cell culture:

Human embryonic kidney 293T cells (HEK293T) were cultured in Dulbecco’s modified Eagle medium (CORNING) supplemented with 10% FBS at 37°C in a 95% O_2_–5% CO_2_ atmosphere. Wild-type cDNAs from rat GluK2 and mutant DNAs were cloned into a pCAG-IRES-EGFP (Addgene plasmid #119739) vector. HEK293T cells were transfected with 1 μg/μl cDNA using the TransIT2020 transfection reagent (Mirus) per 24–48 hrs according to the manufacturer’s instructions and then dissociated using Accutase (Innovative Cell Technologies, Inc). After resuspension, the cells were placed on 35 mm poly-D-lysine-coated dishes (Neuvitro), and electrophysiological records were obtained 4 h later^61^.

#### Insect cell culture:

Sf9 insect cells were cultured in Sf-900 II SFM (Gibco) or HyClone CCM3 cell culture medium (Cytiva) at 27°C. The cells were splited twice a week and used until the 30th passage.

### Electrophysiological recordings

All experiments were conducted in whole-cell configuration from transfected cells. The external solution contained (in mM) 145 NaCl, 2.5 KCl, 1.8 CaCl_2_, 1 MgCl_2_, 5 glucose, and 5 HEPES units. The internal pipette solution contained (in mM) 105 NaCl, 20 NaF, 5 Na_4_BAPTA, 0.5 CaCl_2_, 10 Na_2_ATP, and 5 HEPES units. The pH and osmotic pressure of the external and internal solutions were adjusted to 7.4 and 300–290 mOsmol/Kg, respectively. 1 mM L-glutamate or 10 μM domoate was applied using a theta glass tubing mounted on a piezoelectric stack (MXPZT-300 series, Siskiyou) driven by a HEKA EPC10 amplifier for 1 second to L-glutamate and 10 seconds to domoate. All recordings were performed with HEKA EPC10 amplifiers (HEKA Elektronik, Lambrecht, Germany) using a thin-wall borosilicate glass pipette (2–5 MΩ) coated with dental wax to reduce electrical noise. Currents were recorded with a holding potential of −70 mV, the sampling frequency was 10 kHz, and the signal was filtered at 2.6 kHz. Data acquisition was performed using PULSE software (HEKA Elektronic, Lambrecht, Germany). All experiments were conducted at room temperature (22–24°C).

The microscopic rate of desensitization (tau desensitization) was measured by the exponential fit to the decay of current from ~80% of its peak amplitude (I_peak_) to baseline of recordings where glutamate was applied by 1 second and domoate for 10 seconds taking just the first exponential component of the recording which shown a desensitization kinetics, omitting the steady state current for the domoate activated receptors. The desensitization kinetics were fitted by using the single exponential, one-term fitting (Levenberg-Marquardt). The percentage of desensitization was quantified only to domoate recordings, using the formula:

$$\%\;desensitization=\left[100-\left(\frac{I_{steady-state}}{I_{\text{max}\;peak}}\;*\;100\right)\right]$$

where the I steady state was the maximum current taken at the end of the domoate application. The microscopic rate of deactivation (tau deactivation) was measured from 3 ms glutamate application recordings by exponential fit to the decaying current from ~80% of its peak amplitude to baseline. The deactivation kinetics was fitted by using an exponential 2-terms fitting (Levenberg-Marquardt) for the R775A GluK2mutant. Mean weighted tau deactivation values were calculated using the following formula:

$$\text{tau}\;deactivaion\;=\frac{\left(tau1\;*\;A1)\;-\;(tan2\;*\;A2\right)}{A1\;+\;A2}$$

where tau1 and tau2 are the two terms fitted, A1 is the maxima activation 1 and the A2 is the maxima activation 2. For recovery from desensitization, a paired-pulse protocol was used and the amplitudes of the test pulses were normalized to the amplitude of the desensitizing pulse response (calculated as percentage recovery), and plotted against the log of the interval between desensitizing and test pulses. The time courses of recovery were fitted with one exponential function to calculate the τ recovery.

### Expression and purification of GluK2 KAR

The Sf9 insect cells at 4 × 10^6^ cells/ml were infected with the recombinant baculovirus harboring GluK2_em_ and harvested at 48 h post-infection.

The cells were lysed with a Qsonica sonicator (4 min, 15 s on/off intervals, power level 25–30%) in a buffer containing 50 mM Tris-Cl, pH 8.0, 150 mM NaCl, and 1 mM PMSF. The cell debris was removed with 20 min centrifugation at 6,500 g followed by ultracentrifugation at 180,000 g buffer containing 50 mM Tris-Cl pH 8.0, 150 mM NaCl, and 15 mM dodecyl-β-D-maltoside (DDM, Anatrace) for 2 h at 4°C and centrifuged at 180,000 g for 45 min. The Streptactin XT sepharose resin (1.0–1.2 ml per liter culture) was packed into glass columns, and the solubilized proteins were bound using an enhanced gravity flow set at approximately 0.5 ml/min. The unbound proteins were washed with 15 CV of the wash buffer (lysis buffer supplemented with 2 mM DDM) and eluted in 5–8 fractions of approximately 1 CV each with the elution buffer (lysis buffer supplemented with 2 mM DDM and 50 mM biotin). Subsequently, the eluted GluK2 sample was concentrated and loaded onto a Superose 6 increase 10/300 (GE Healthcare) column pre-equilibrated with 50 mM Tris-Cl pH 8.0, 150 mM NaCl, and 1 mM DDM. The peak fractions were pooled for cryo-EM specimen preparation and concentrated at approximately 4 mg/ml. All steps were performed at 4°C.

### Mass spectroscopy

For protein digestion, the bands were cut from the gel with a punch machine and washed/destained in 50% ethanol and 5% acetic acid. The gel pieces were then dehydrated in acetonitrile, dried in a Speedvac chamber, digested with trypsin by adding 5 μl of 10 ng/ μl proteases in 50 mM ammonium bicarbonate, and incubated overnight digestion at room temperature. The peptides that were formed were extracted from the polyacrylamide in two aliquots of 30 μl each, with 50% acetonitrile with 5% formic acid. These extracts were combined and evaporated to <10 μl in a Speedvac chamber and then resuspended in 1% acetic acid to form a final volume of approximately 30 μl for the LC-MS analysis. The LC-MS system used was a Finnigan LTQ-Obitrap Fusion Lumos hybrid mass spectrometer. The HPLC column was a Dionex 15 cm × 75 μm id Acclaim Pepmap C18, 2 μm, 100 Å reversed-phase capillary chromatography column. Five microliters of the extract were injected, and the peptides eluted from the column by using an acetonitrile/0.1% formic acid gradient at a flow rate of 0.25 μl/min were introduced into the source of the mass spectrometer online. The digest was analyzed using the data-dependent multitask capability of the instrument by acquiring full-scan mass spectra to determine peptide molecular weights and product ion spectra to determine the amino acid sequence in successive instrument scans. These data were searched spectra specifically against the rat GluK2 sequencing using all CID or EThcD spectra collected in the experiment to search specifically against the proteins of interest with the search programs Sequest (CID) or MSFragger (EThcD) program considering N-linked glycosylation as a variable modification.

### TEM sample preparation and data collection

The protein samples were vitrified on glow-discharged UltrAufoil holey-gold film grids (Quantifoil) using FEI Vitrobot Mark IV at 4°C and 22°C, respectively, at 100% humidity, with a blot time of 2–8 s under level 10 blot force.

PELCO easiGlow^™^ was used to render all grids hydrophilic by glow discharging for 60 s at 30 mA. In addition, 2–4 μl of the receptor sample (~4 mg/ml) was applied onto the grid. All micrographs were acquired using Titan Krios (FEI) at the Stanford-SLAC Cryo-Electron Microscopy Facility at 300 kV and the GATAN K3 Summit direct electron detector coupled with the GIF quantum energy filter (Gatan Inc.) at 81,000 magnification in super-resolution mode (0.53/1.06 Å/pixel), with the defocus range of −1.0 to −2.0 μm, and over 50–60 frames and 2.5–5 s exposure, yielding a total dose of 60 e^−^/Å^2^. Automated data collection was conducted using EPU software (Thermofisher Scientific).

### Data processing

The movie alignment and subsequent data processing were performed using Cryosparc v3.2 and 3.31 (Structura Biotechnology Inc.) with various wrappers. CTF estimation was performed using wrapper Gctf 1.06, and the initial particles were selected automatically. The final particle selection was performed using the Topaz particle picker [8]. The selected particle images were subjected to several rounds of 2D classifications to remove misaligned and damaged particles. The selected particles were subjected to iterative rounds of homogeneous and nonuniform refinements using an imported reference map (EMD-8289) [2] low-passed to 30 Å. Subsequent heterogeneous refinement was performed to classify the particles in distinct 3D classes with C1 symmetry. The 3D classes were inspected using UCSF Chimera, and the particles from similar 3D classes were merged, further refined, and classified. The 3D classes that did not contain density for the TMDs were excluded from further refinement. If the 3D classes looked symmetrical, C2 symmetry was imposed, and the particles were further refined using per-particle CTF refinement and B-factor sharpening. The final map was refined using a standalone program deepEMhancer. Model building was initially performed by docking the structural coordinates of GluK2 experimental cryo-EM density maps using UCSF ChimeraX^65,66^. The resulting models were manually inspected using Coot, where bond lengths and torsion angles were corrected to accommodate the missing sidechains and loops and the rotamer orientations were corrected to avoid steric clashes. The prepared structure was further modified to fit into the experimental density map using COOT^67^. The final models were refined against the cryo-EM maps using Phenix real-space refinement ^68^ with a secondary structure and Ramachandran restraints.

## Figures and Tables

**Key source table:** 

				
Reagent type (species) or resource	Designation	Source or reference	Identifiers	Additional information
Gene (*Rattus norvegicus*)	GRIK2_RAT	Genscript (synthesized)	P42260	
Cell line (*Homo sapiens*)	HEK293S GnTI-	ATCC	Cat. No. CRL-3022	
Cell line (*Homo sapiens*)	HEK293T	ATCC	Cat. No. CRL-3216	
Cell line (insect)	Sf9	Novagen	Cat. No. 71104	
Chemical compound, drug	L-glutamate	TCI Chemicals	Cat. No. G0188	
Chemical compound, drug	Domoate	Sigma	Cat. No. D6152	
Software, algorithm	Pulse	HEKA	https://scicrunch.org/resolver/SCR_018399	https://www.heka.com/
Software, algorithm	OriginPro 2020	OriginLab	RRID:SCR_014212	https://www.originlab.com/2020
Software, algorithm	Relion 3.1, Relion 4.0 beta	doi:10.7554/eLife.42166	RRID:SCR_016274	https://www3.mrc-lmb.cam.ac.uk/relion/index.php/Main_Page
Software, algorithm	cryoSPARC 3.x	doi:10.1038/nmeth.4169.	RRID:SCR_016501	https://cryosparc.com/
Software, algorithm	GCTF	doi:10.1016/j.jsb.2015.11.003	RRID:SCR_016500	https://www2.mrc-lmb.cam.ac.uk/download/gctf_v1-06-and-examples/
Software, algorithm	CTFFIND 4.1	doi:10.1016/j.jsb.2015.08.008	RRID:SCR_016732	http://grigoriefflab.janelia.org/ctffind4
Software, algorithm	UCSF Chimera	doi:10.1002/jcc.20084	RRID:SCR_004097	http://plato.cgl.ucsf.edu/chimera/
Software, algorithm	UCSF ChimeraX	doi:10.1002/pro.3943	RRID:SCR_015872	https://www.cgl.ucsf.edu/chimerax/
Software, algorithm	HOLE	doi:10.1016/s0263-7855(97)00009-x	NA	http://www.holeprogram.org
Software, algorithm	COOT 0.9x	doi:10.1107/S0907444910007493	RRID:SCR_014222	http://www2.mrc-lmb.cam.ac.uk/personal/pemsley/coot/
Software, algorithm	Phenix 1.20	doi:10.1107/S2059798319011471	RRID:SCR_014224	https://www.phenix-online.org/
Software, algorithm	MolProbity	doi:10.1107/S0907444909042073	RRID:SCR_014226	http://molprobity.biochem.duke.edu
Software, algorithm	deepEMhancer	https://doi.org/10.1038/s42003-021-02399-1	NA	https://github.com/rsanchezgarc/deepEMhancer
Software, algorithm	Topaz	https://doi.org/10.1038/s41592-019-0575-8	NA	https://github.com/tbepler/topaz
Software, algorithm	ISOLDE	doi:10.1107/S2059798318002425	NA	https://isolde.cimr.cam.ac.uk/

## Data Availability

Cryo-EM density maps and atomic coordinates for GluK2-apo and GluK2-domoate were deposited in the electron microscopy data bank under the accession codes listed below.

## Highlights

1. High-resolution structures of kainate-type ionotropic glutamate receptors.
2. Comparison of apo- and partial agonist bound homomeric GluK2 kainate receptors illuminates conformational transitions.
3. Intact GluK2 kainate receptors in the apo ligand-free state are stabilized in the desensitized conformation in detergent micelles.
4. Identification of a gating switch of KARs on desensitization.
5. Identification of *N*-glycosylation involved in KAR gating.
